# Plasmonic Optical Imaging of Gold Nanorods Localization in Small Animals

**DOI:** 10.1038/s41598-018-27624-6

**Published:** 2018-06-19

**Authors:** Keying Xu, Junwei Shi, Ali Pourmand, Thirupandiyur S. Udayakumar, Nesrin Dogan, Weizhao Zhao, Alan Pollack, Yidong Yang

**Affiliations:** 1 Department of Radiation Oncology, University of Miami School of Medicine, Miami, FL 33136 USA; 2 Department of Biomedical Engineering, University of Miami College of Engineering, Coral Gables, FL 33146 USA; 3 Department of Marine Geoscience, University of Miami RSMAS, Miami, FL 33149 USA

## Abstract

Gold nanoparticles (GNP) have been intensively investigated for applications in cancer imaging and therapy. Most imaging studies focused on microscopic imaging. Their potential as optical imaging probes for whole body small animal imaging has rarely been explored. Taking advantage of their surface plasmon resonance (SPR) properties, we aim to develop a noninvasive diffuse optical imaging method to map the distribution of a special type of GNP, gold nanorods (GNR), in small animals. We developed an integrated dual-modality imaging system capable of both x-ray computed tomography (XCT) and diffuse optical tomography (DOT). XCT provides the animal anatomy and contour required for DOT; DOT maps the distribution of GNR in the animal. This SPR enhanced optical imaging (SPROI) technique was investigated using simulation, phantom and mouse experiments. The distribution of GNR at various concentrations (0.1–100 nM, or 3.5 ug/g–3.5 mg/g) was successfully reconstructed from centimeter-scaled volumes. SPROI detected GNR at 18 μg/g concentration in the mouse breast tumor, and is 3 orders more sensitive than x-ray imaging. This study demonstrated the high sensitivity of SPROI in mapping GNR distributions in small animals. It does not require additional imaging tags other than GNR themselves. SPROI can be used to detect tumors targeted by GNR via passive targeting based on enhanced permeability and retention or via active targeting using biologically conjugated ligands.

## Introduction

Because of their favorable chemical, physical and biological properties, gold nanoparticles (GNP) have been intensively investigated for broad applications in medicine. These applications include, but not limited to, biosensing^1–4^, cell imaging^5,6^, cell labeling^7,8^, drug delivery^9–12^, cancer imaging^13–16^, and cancer therapy^17–20^. Because of the “surface plasmon resonance” (SPR) phenomenon, GNP can drastically absorb and scatter light matching their resonance wavelengths, up to 10^5^ times more than the conventional dyes, quantum dots or fluorescent agents do^21^. The peak absorption/scattering wavelengths are determined by the size, shape and internal structure of a particular type of GNP^21–23^. Additional advantages of GNP include good biocompatibility, facile synthesis and easy conjugation with various targeting ligands. These favorable features have made GNP an ideal candidate in cellular optical imaging^24–27^ and photothermal cancer treatment^28–30^. Gold nanorods (GNR) are rod-shape GNP with flat, elliptical or hemispherical ends^31^. Of particular interest, their aspect ratio (ratio of length to diameter) determines the resonance wavelength, and can be tuned during production to match light of different wavelengths^29^.

Noninvasive *in vivo* imaging of GNP can be of tremendous value in cancer diagnosis and treatment evaluation where GNP serves as targeting probes. Since GNP have higher density and atomic number than human tissues and consequently higher x-ray attenuation, x-ray computed tomography (XCT) can be a potential modality for GNP imaging. However this requires high GNP concentration of at least tens of milligram per gram (mg/g)^27,32^. Within the energy range of diagnostic x-ray (~10–120 keV), the photoelectric effect dominates the interaction between photons and GNP where GNP absorbs photon energy and produces characteristic x-ray which is also often called x-ray fluorescence. Researchers have developed x-ray fluorescence imaging for *in vivo* GNP detection. But such method requires a high x-ray dose to effectively excite adequate x-ray fluorescence signal for tomography reconstruction^33–38^. Another strategy for GNP detection is conjugating them with paramagnetic material such as gadolinium (Gd) and iron (Fe), which makes them detectable in magnetic resonance imaging (MRI)^39–42^. However, this is an indirect detection process which not only requires non-trivial particle synthesis but also has limited sensitivity.

Diffuse optical tomography (DOT) is a non-invasive imaging technique that reconstructs the three dimensional (3D) distribution of the optical properties of an imaged object. In DOT, near infrared (NIR) light in 650–900 nm wavelength range is usually used as the light source due to their deep tissue penetration^27,29^. Fiber-coupled optical detectors or simply a charge-coupled detector (CCD) camera are used to detect the light transmission. The optical properties, i.e., the absorption and scattering coefficients, can be inversely calculated by mathematically solving the light diffusion equation. However, the reconstruction algorithm for DOT is a well-known ill-posed mathematical problem where the unknowns in the problem are much more than the knowns. In addition, the diffuse pattern of the optical light propagation in the tissue causes further blur of reconstructed objects. Therefore the resolution of DOT is typically in the order of millimeter in small animal imaging, which is comparable to Positron Emission Tomography (PET) and Single Photon Emission Computed Tomography (SPECT), and inferior to Computed Tomography (CT) and MRI. Nonetheless, comparing to other imaging modalities, DOT has advantages in its fast image acquisition, high imaging throughput, and low instrumentation cost^43^. DOT has great potential for noninvasive imaging of GNP. First, GNP have much higher light attenuation than normal tissues attributing to the SPR phenomenon^29^; second, GNP can accumulate to higher concentration in tumors than they do in surrounding tissues through the enhanced permeability and retention effect^44–46^, or through active targeting strategy where GNP are conjugated with cancer specific antibodies or ligands^9,29^. Previous studies have used various optical imaging techniques for GNP detection. Two-dimensional diffuse optical imaging and diffuse optical spectroscopy have been used to detect GNP in phantom^47–50^. Optical coherent tomography (OCT) has also been explored for GNP detection^51–54^. However, these optical images were obtained either with no depth dimension or with very superficial coverage. In addition, most optical imaging studies focused on microscopic imaging at micrometer and nanometer scales^55–58^. The DOT in this contribution is particularly referred to the optical imaging method that reconstructs the optical properties of imaged subject from transmission optical projections, and distinguishes from the emission-based optical imaging methods such as bioluminescence and fluorescence tomography^59–63^. To the best of our knowledge, this is one of the first studies that investigated transmission-based whole body DOT of centimeter-scaled small animals with GNR as contrast agents.

We aim to develop a noninvasive SPR enhanced optical imaging/tomography (SPROI/SPROT) technique to map GNR distribution in small animals. We first built an integrated XCT/DOT imaging system for dual-modal GNR imaging. XCT was used to acquire the animal anatomy. It also creates the volumetric mesh needed in SPROT tomographic reconstruction. SPROT was first performed in simulation as a proof of concept, then on a tissue-simulating phantom to test the capability of our imaging system in GNR mapping, and finally on tumor-bearing mice to demonstrate its feasibility for whole body small animal imaging.

## Results

### Simulation

**Figure 1:**
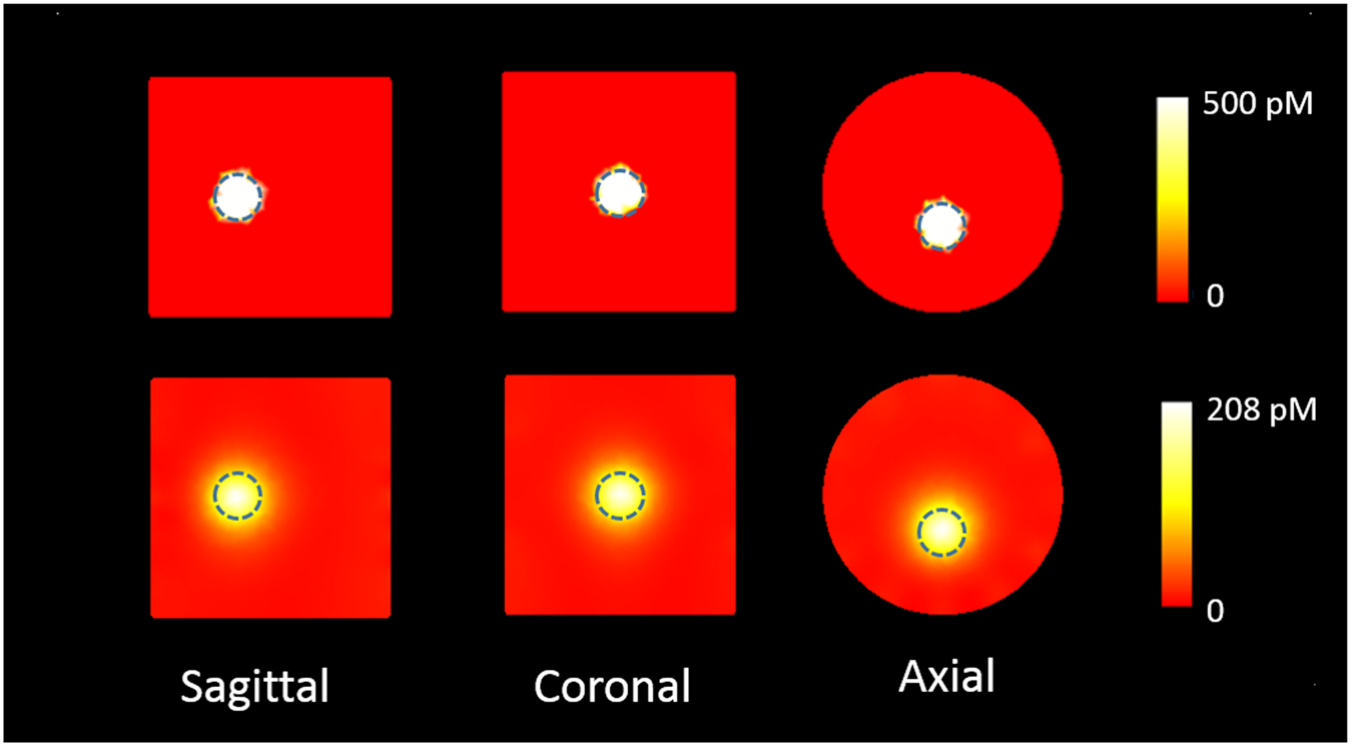
The DOT reconstruction in the simulation study. The top row shows the ground truth, the bottom row the reconstructed GNR distribution. The dashed circles outline the ground truth boundaries of the GNR volume. The reconstruction resulted in expanded GNR distribution and hence reduced concentration.

Figure 1 presents the SPROT reconstruction results from the simulation study for the GNR volume at 500 pM concentration. The top row shows the true GNR distribution, with the center of mass (COM) localized at (0, −4, 0) mm. The bottom row shows the reconstructed distribution. The reconstructed maximum concentration located at (−0.1, −4.2, 0.0) mm (off by 0.2 mm radial distance). The GNR concentration was interpreted from the reconstructed absorption coefficient. The maximum concentration was recovered as 208 pM instead of 500 pM (ground truth). GNR distribution was also successfully reconstructed for all three other concentrations at 1 nM, 200 pM and 100 pM levels. The recovered maximum concentrations were 0.34 nM, 89.4 pM, and 51.4 pM, and their locations were 0.4, 0.3, and 0.5 mm off the ground truth, respectively. The simulation results indicate the high sensitivity of SPROT, which can detect GNR at sub-nanomolar concentration level (<3.71 µg/g) with submillimeter spatial accuracy.

### System Calibration

#### CCD Camera Performance

**Figure 2:**
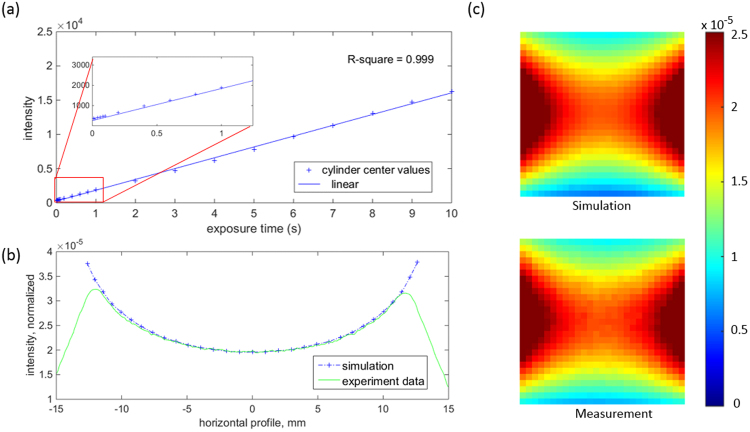
The camera reading linearity and light fluence calibration results. The linear curve fitting (R^2^ = 0.999) in (**a**) indicates the light intensity is proportional to the exposure time. (**b**,**c**) Demonstrate a good match between the simulation and measurement data. (**b**) Shows the horizontal profile across the center of the light filed. (**c**) Shows the light distributions (1 mm resolution) measured and simulated respectively within an central region of interest of 27 mm × 27 mm.

Image distortion was deemed negligible (less than 1 pixel) across the field of view (FOV). It was not detectable at the center 30 × 30 mm^2^ area where measurement data was extracted for SPROT reconstruction. The image intensity decreased by 50% at the 4 corners of the FOV, but only by 5% within the center 30 × 30 mm^2^ area. Light intensity across the raw image was compensated through multiplication of the uniformity map. The dark current and readout noise measured together was 287.84 ± 2.40 (averaged over 10 images, 1024 × 1024 pixels per image) while the total offset was 287.87 ± 2.40. The negligible difference indicates minimal light contribution from the background light. The light intensity was linearly proportional to the exposure time (R^2^ = 0.999), as shown in Fig. 2(a).

#### Light Fluence Calibration

A normalization factor of 5.63 × 10^−10^ was obtained through the calibration procedure and applied to the projection images in phantom experiment. Figure 2(c) shows the comparison between the simulation and calibrated measurement data. A good agreement was observed and further confirmed with the profiles drawn across the center of light (Fig. 2(b)). The average difference (calculated as the percentage difference of signal intensity for each pixel) across the center region of interest (ROI) (22 mm × 18 mm) was 1.59%. This result validated our fluence calibration procedure.

### Phantom Study

**Figure 3:**
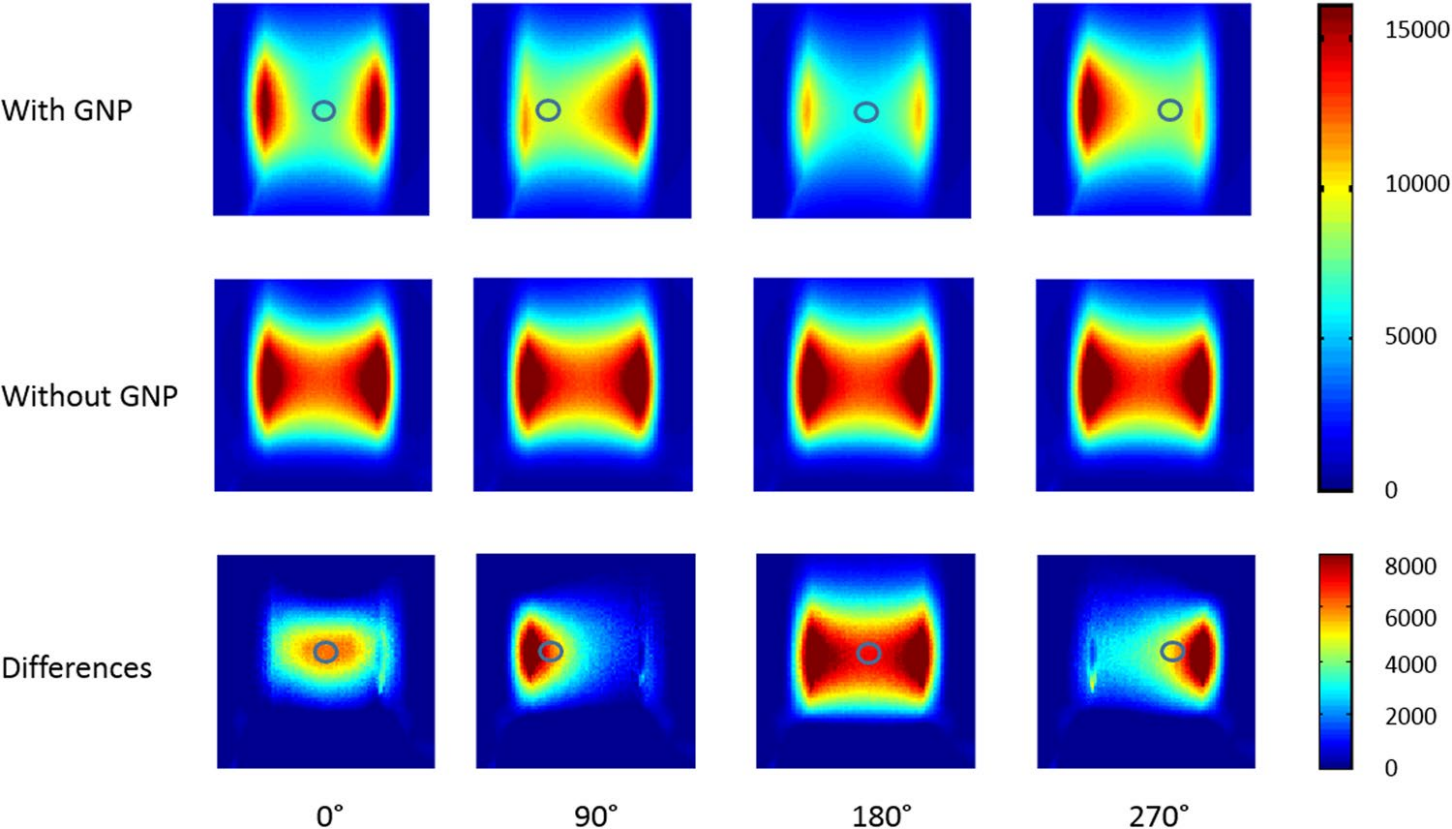
Projection optical images at 0°, 90°, 180° and 270°, respectively. Circles on the projections indicate the projected GNR locations. The third row shows the differences between projections with and without GNR, indicating the amount of light absorbed by GNR.

**Figure 4:**
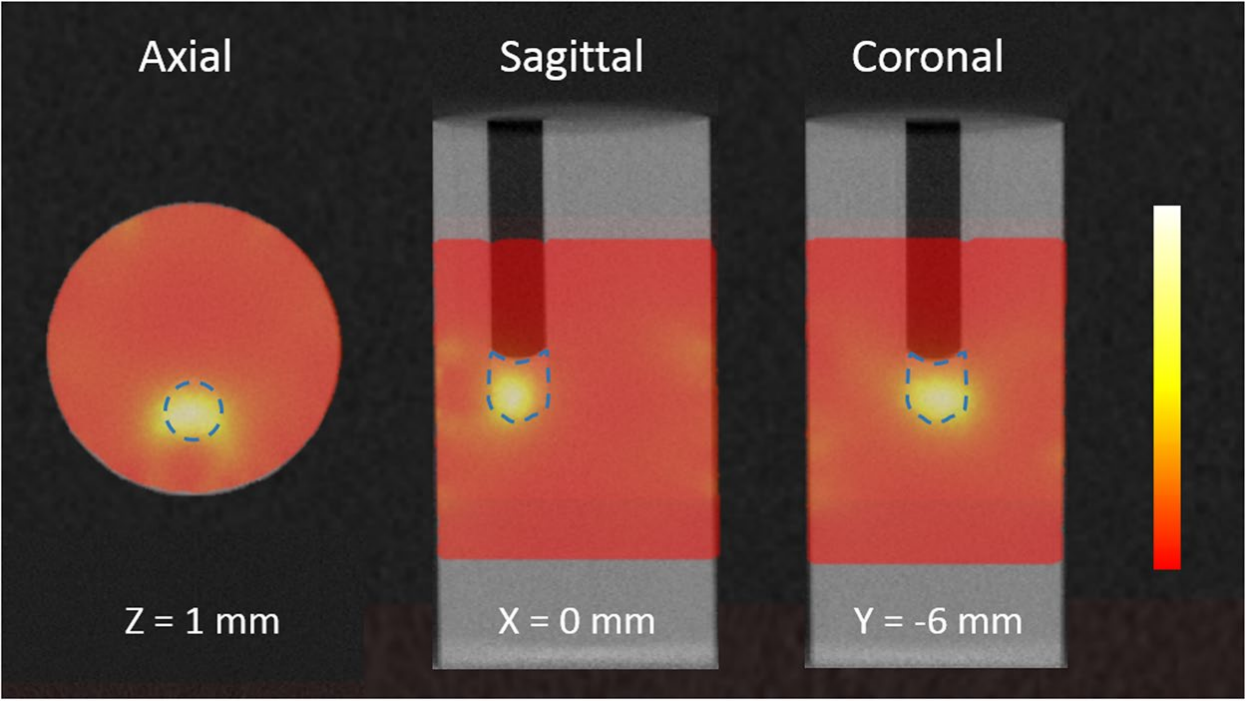
The SPROT images Superimposed on XCT for the GNR-containing phantom. Dashed lines mark the ground truth GNR volume. The GNR volume in SPROT co-localized with that in XCT, but showed a broader distribution. The average GNR concentration in SPROT was ~1 nM. Image artifacts are also observed in the phantom background.

Figure 3 shows projection images from 4 different angles. Image intensity was smaller for the GNR group than the non-GNR group, due to the strong light absorption. The differences between projections with and without GNR indicate the amount of light absorbed by GNR. The SPROT images are superimposed on XCT images in Fig. 4. The two reconstructed GNR volumes from SPROT and from XCT co-localized with each other. The COM of the GNR volume in SPROT deviated from that in XCT by 2.0 mm. The shape of the GNR volume reconstructed in SPROT differed from its ground truth as seen in XCT, and showed a broader distribution. The average GNR concentration recovered by SPROT was ~1 nM which was lower than the ground truth value (96.3 nM). Low intensity image artifacts are also observed in the phantom background in SPROT images.

### Animal Study

**Figure 5:**
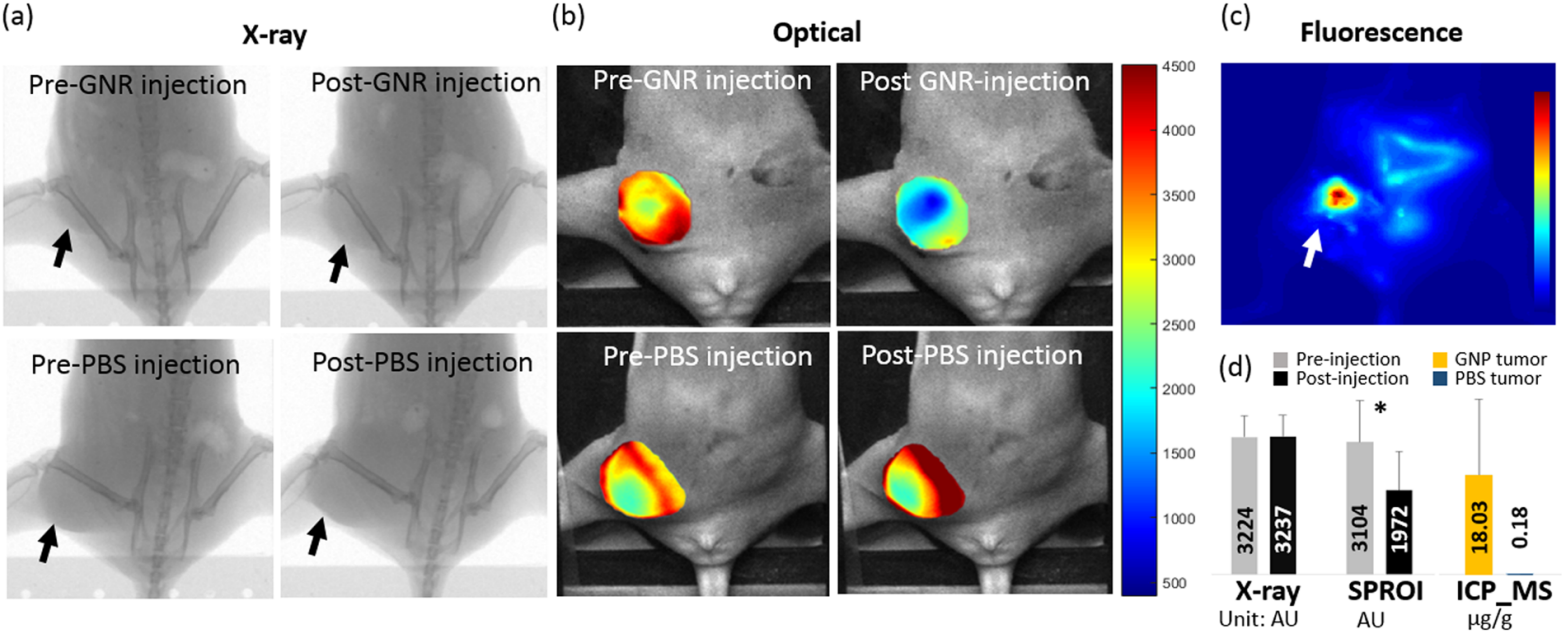
*In Vivo* imaging and ICP-MS results. X-ray (**a**) and optical images (**b**) before and after intra-tumor injection of 40 μl GNR (upper) or PBS (lower). While post-injection x-ray shows no enhancement at tumor site, dramatic signal decrease in post-GNR injection optical image indicates strong light absorption at the tumor site. No significant difference is shown in PBS-injection images. (**c**) The fluorescence image taken 2 hours after GNR injection shows strong fluorescence coming from the GNR in the tumor site. (**d**) The statistical analysis result comparing the signal intensity in the x-ray and optical images (n = 4) before and after GNR injection. It also shows the ICP-MS result for 3 tumors harvested after GNR injection and 1 tumor after PBS injection. *Indicates a significant difference at p = 0.004. The black and white arrows point to the tumor site. AU is for arbitrary unit.

Figure 5 shows the x-ray and optical images pre- and post- GNR injection into the tumor for one animal. While there was no visible change in the x-ray images for all animals (n = 4, p = 0.853), the light transmission in the tumor region decreased significantly in the optical images (n = 4, p = 0.004) after intra-tumor GNR injection, see Fig. 5(a,b,d). Meanwhile, the light transmission was not reduced for the control mouse that received intra-tumor phosphate-buffered saline (PBS) injection. Fluorescence image taken 2 hours after injection confirmed the existence of GNR in the tumor region. Low-intensity fluorescence was also observed in the abdomen, indicating some GNR were already metabolized through gastrointestinal tracks at that time. Inductively coupled plasma mass spectrometry (ICP-MS) test reported a gold concentration of 18.03 ± 13.82 μg/g (equivalent to 0.49 ± 0.38 nM GNR, n = 3) in the harvested tumors for the GNR group, compared to 0.18 μg/g for the control group. The lowest gold concentration was 9.41 μg/g based on the ICP-MS analysis of one harvested tumor.

**Figure 6:**
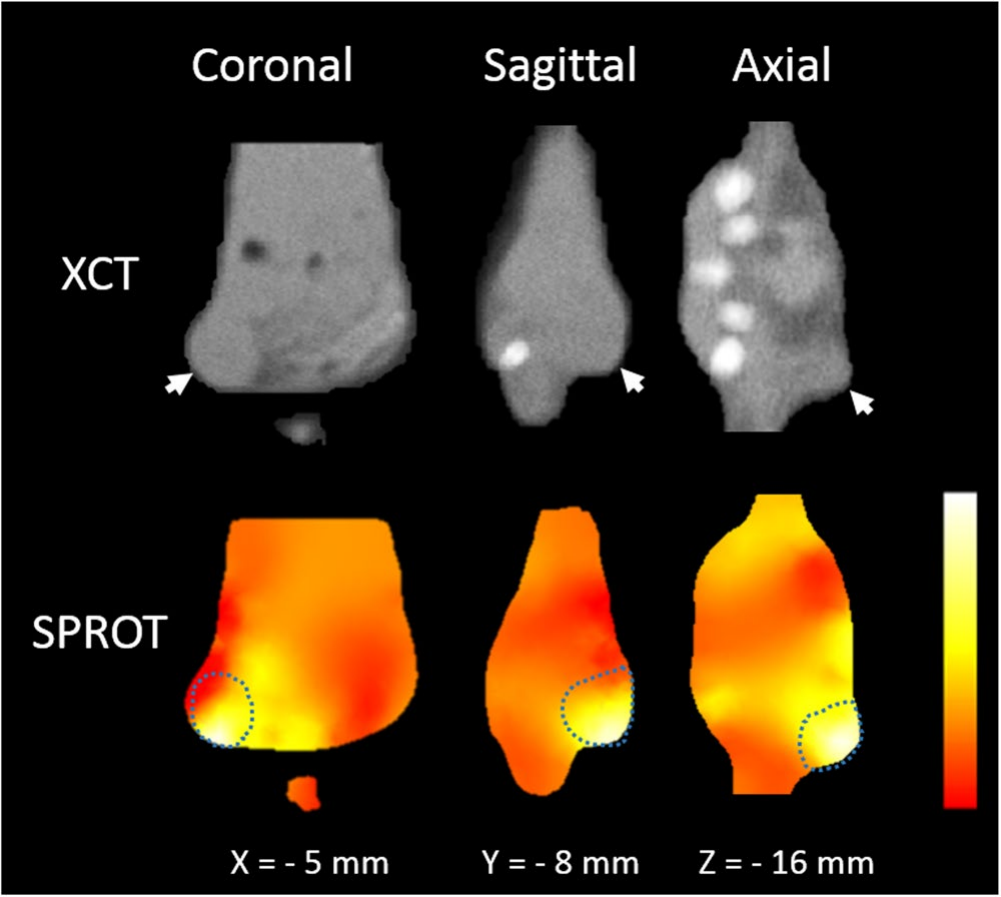
*In vivo* XCT and SPROT. Arrows on XCT images point to tumor location identified by tumor morphology. No image contrast was observed between the GNR-containing tumor and back ground tissues in XCT images. The dashed lines in SPROT mark the tumor contour determined from XCT. High-concentration GNR distribution was restored inside the tumor volume. The medium-intensity signal nearby the tumor may be caused by diffused GNR after intra-tumor injection.

The SPROT results are shown in Fig. 6 in three orthogonal views. High concentration of GNR distribution was recovered inside the tumor volume. Due to limited quality of tomographic optical reconstruction, low intensity signal was also observed in background. The signal nearby the tumor, however, may be caused by the diffusion of nanoparticles after injection.

## Discussion

Spatial and quantitative accuracy is always a challenge for DOT imaging. DOT reconstruction is a renowned ill-posed mathematical problem, due to the overwhelmed amount of unknown variables. Specifically, the absorption and scattering coefficients cannot be reliably decoupled in the continuous-wave based DOT imaging^64^. In this study, laser light of a single wavelength was used as the light source. To reduce the freedom of reconstruction, we took advantage of the known relationship that exists between the absorption and scattering coefficients of the GNR. For voxels that were not recognized as GNR-containing volume the scattering coefficients were fixed at their initial values during iterations of reconstruction. In this manner, the number of unknown variables was reduced to half. The absorption coefficient for the abdominal mouse tissue was assigned as 0.01 mm^−1^, which is equivalent to 41pM GNR. Considering that the absorption of GNR is greater than that of mouse tissues, this reconstruction strategy is expected to recover GNR-containing volume that bears a GNR concentration of >> 41 pM. In the future, multi-wavelength imaging technique may be used to improve the SPROT reconstruction.

Jacobian normalization was implemented to mitigate the steep optical property gradient between neighboring nodes, and to yield to a smooth and relatively continuous GNR distribution. However, this strategy may cause over-estimation of GNR-containing tumor volume and under-estimation of GNR concentration. It is worth to mention that this is the very first *in vivo* study of SPROT imaging method, and our current study focused on qualitatively imaging GNR distribution. Quantitative imaging studies with absolute GNR concentration assessment will be investigated in the future along with the development of better SPROT reconstruction algorithms.

*In vivo* SPROT in the animal faces specific challenges. Organs such as gastrointestinal tracks absorb light strongly and thus cause artifacts in the reconstructed image of GNR distribution. In this study, projection images after and before GNR administration were subtracted to distinguish the net signal caused by GNR attenuation. Hence we minimize the image artifacts caused by highly-attenuating organs. The subtraction method, however, requires a good registration of two corresponding projection images, which requires high reproducibility of animal positioning and imaging setup.

GNP can be indirectly imaged when they are combined with other imaging agents. For example, magnetic nanoparticles (gadolinium or iron oxide nanoparticles) can be combined with GNP for *in vivo* MRI imaging^39–42^. Fluorophores can also be conjugated with GNP for *in vivo* fluorescence imaging^27^, as we did in our validation study. These, however, require additional synthetic procedures to effectively combine GNP and other imaging agents. These procedures are non-trivial and sometimes very challenging (e.g., conjugating fluorophores to GNP requires a precise control of the length of the linker to avoid fluorescence quench by GNP). In contrast, SPROI does not require additional imaging tags attached to GNR. It takes the advantage of SPR effect which is an inherent property of metal nanoparticles, and does not need additional conjugation. It can be used to detect tumors targeted by GNR, either via passive targeting based on enhanced permeability and retention or via active targeting using biologically conjugated ligands.

In the current imaging study GNR was administrated via intra-tumor injection. While this is a convenient way to demonstrate the feasibility of GNR enhancement, intravenous administration may be a more appropriate delivery route, particularly for internal tumors deep in the body^13,32,42^. In such cases, the pharmacokinetics and pharmacodynamics of GNR is affected by their size, shape, and surface modification. Although gold is chemically inert, numerous toxicity studies suggest that their biodistribution and toxicity should be considered on a case by case manner^11,65^.

## Conclusion

The concept of SPROI imaging was validated on an in-house developed dual-modality XCT/DOT imaging platform. GNR of various concentrations (0.1–100 nM) was successfully reconstructed from centimeter-scaled volumes in simulation, phantom and animal experiments. SPROI detected GNR at a concentration as low as 9.41 μg/g, which is too low to be detected in x-ray imaging. This study demonstrated the high sensitivity of SPROI in mapping GNR distributions in small animals. SPROI does not require additional imaging tags other than GNR themselves. It can be used to detect tumors targeted by GNR, either via enhanced permeability and retention effect or using biologically conjugated ligands.

## Materials and Methods

### Gold Nanoparticles and Phantoms

We used GNR for all experiments in this study. They had 10 nm diameter and 45 nm length with an aspect ratio of 4.5 (Nanopartz Inc., Loveland, CO, USA). The peak light extinction occurred at 850 nm wavelength, and the manufacturer measured absorption coefficient was 23.49 mm^−1^ at 96.3 nM concentration. The theoretical values of peak absorption and scattering cross-section is 1.15 × 10^−15^ m^2^ and 2.27 × 10^−17^ m^2^, respectively, assuming random orientation of GNR alignment. The GNR were also conjugated with Cy5 fluorophore (excitation 650 nm, emission 670 nm) which allows fluorescence imaging. The phantom was a 30 mm-diameter and 60 mm-height cylinder mimicking mouse size and made of tissue-simulating material in the near-infrared optical range (INO Inc., Quebec City, Quebec, Canada; absorption coefficient µ_a_ 0.01 mm^−1^ and reduced scattering coefficient µ_s_^’^ 1.01 mm^−1^ at 856 nm). A cylindrical hole of 6 mm diameter and 32 mm depth was drilled parallel to but 6 mm away from the central axis to contain GNR colloid. A cuboid phantom of 30 mm × 30 mm × 20 mm size made of the same material was used during the procedure of optical system calibration.

### Simulation

Forward light propagation and inverse optical image reconstruction were performed on NIRFAST, an open source optical imaging software platform^66,67^. We adapted the NIRFAST reconstruction algorithm for more consistent optimization outcome. Simulation was performed on a homogeneous cylindrical digital phantom of 30 mm height and 30 mm diameter which had an absorption coefficient of 0.01 mm^−1^ and reduced scattering coefficient of 1.0 mm^−1^. The following GNR parameters were used: peak attenuation at 850 nm for the longitudinal SPR; absorption coefficient 23.49 mm^−1^ at 96.3 nM concentration; calculated absorption to scattering ratio of 50. A 6 mm-diameter sphere containing GNR was placed inside the digital phantom. Simulations were performed at four concentrations: 1 nanomolar (nM), 500 picomolar (pM), 200 pM and 100 pM (6.02 × 10^11^, 3.01 × 10^11^, 1.20 × 10^11^ and 6.02 × 10^10^ particles/ml, respectively). The light source and detector points were placed 30° apart circumferentially (12 points at each row) and 2.5 mm apart longitudinally (9 rows). When one point was assigned the source, all the points on the opposite side of the phantom that were within 5 mm longitudinally and 120° circumferentially relative to the projection point of the source became detectors. Hence there were 108 source points in total and 25 detector points corresponding to each source. Two high resolution 3D cylindrical meshes (0.5 mm node-to-node distance, 33186 and 33029 nodes, respectively, for the forward and reconstruction mesh) were generated from the digital phantom. Forward light transport was run at 850 nm wavelength, and 2% Gaussian noise was added to the light transmission data before it was used for reconstruction. SPROT Reconstruction process took 35 minutes on a computer with 3.4 GHz CPU and 32GB RAM.

### XCT/DOT Imaging Platform

**Figure 7:**
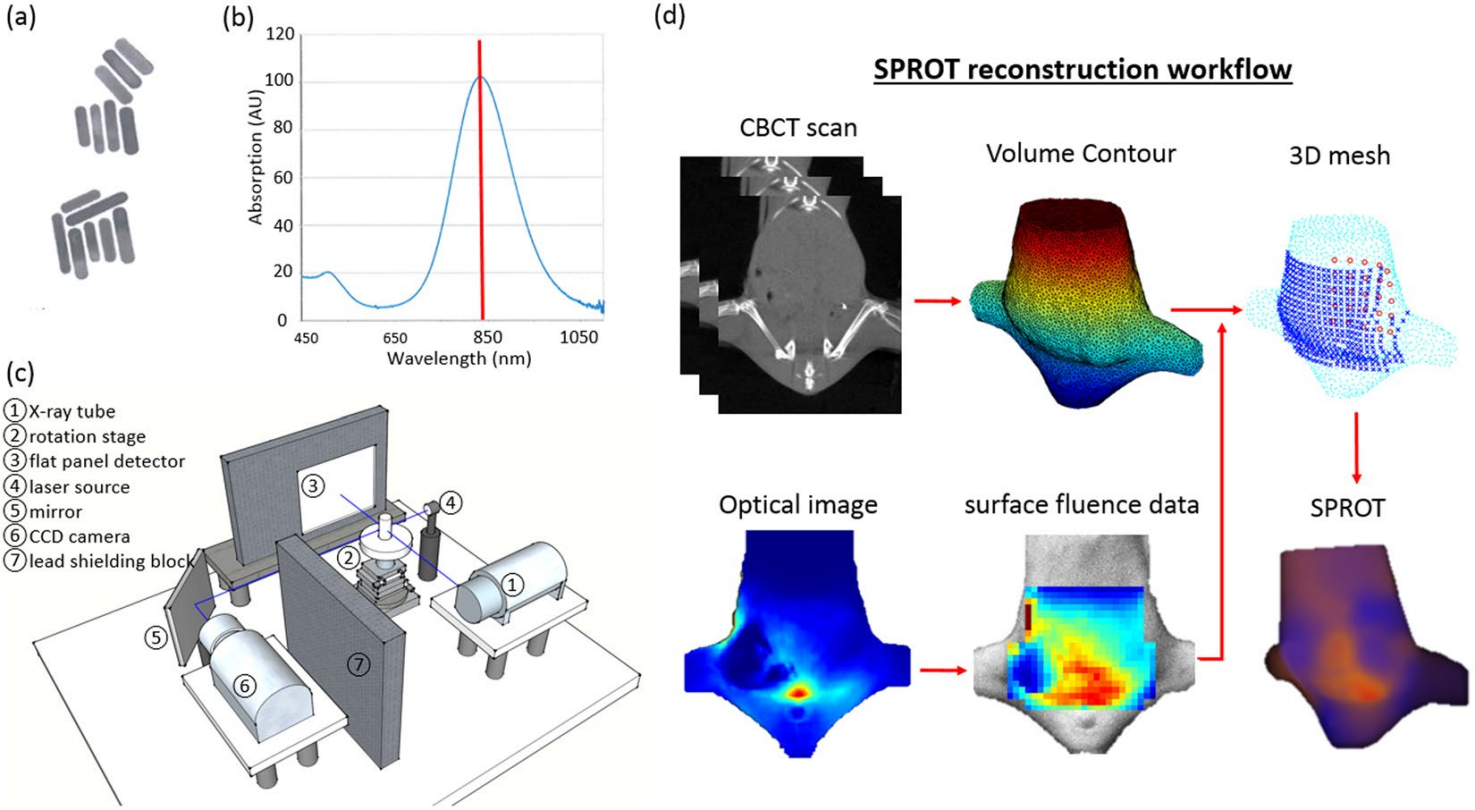
The GNR, imaging system and reconstruction workflow for SPROT imaging. (**a**) GNR had 10 nm diameter and 45 nm length, leading to peak absorption at 850 nm (**b**). (**c**) The integrated XCT/DOT system. The XCT system consists of an x-ray source, a rotation stage and a flat panel detector. The DOT system consists of a laser source whose trajectory projects through the XCT image isocenter, a 45° mirror and a lens-coupled CCD camera. A lead block stands between the camera and XCT assembly to shield the scattered x-ray. (**d**) SPROT reconstruction workflow. XCT scan was used to acquire the object volume for 3D mesh generation. Optical images were processed to extract surface fluence data which was then mapped to the 3D mesh. The 3D mesh, surface fluence data and locations of laser source were input for SPROT reconstruction.

The configuration of our in-house developed XCT/DOT imaging platform is shown in Fig. 7(c). The dual-modality imaging system was built in a dark enclosure that shielded both internal radiation and external room light. For DOT imaging, laser light of 856 nm wavelength, close to the peak absorption wavelength (850 nm) of the GNR, was generated from a fiber-coupled four channel laser source (Thorlabs, Newton, NJ, USA) and collimated to a 1 mm focal spot projected onto the surface of the imaged object. The laser generator was placed outside the enclosure to reduce background light. The animal stage was capable of continuous rotation and x-y-z translation. A CCD camera (Andor, Concord, MA, USA) with a 13.3 mm × 13.3 mm sensor and 1024 × 1024 pixels coupled with an f/1.8 lens (Nikon, Minato-ku, Tokyo, Japan) was positioned on the opposite side of the laser source to capture the light transmitted through the imaged object. The work distance from the lens to the object was 45 cm. The pixel resolution at the image plane was 0.195 mm and FOV was 87 × 87 mm^2^.

The XCT was initially developed to provide image guidance for small animal radiation^68^. It employed an x-ray tube (COMET AG, Flamatt, Switzerland), an amorphous silicon (a-Si) flat panel detector (PerkinElmer, Waltham, MA, USA), and the rotation stage shared with the DOT system. The animal was positioned upright and rotated around its Cephalocaudal axis, between the stationary x-ray source and detector panel. The source to detector distance and source to rotation axis distance were 53.7 cm and 34.2 cm, respectively, resulting in an image magnification of 1.57 at the image panel. The x-ray tube had a nominal voltage up to 225 kVp and dual focal spots of 1.0 mm and 5.5 mm (EN12543 standard) corresponding to continuous ratings of 640 W and 3000 W, respectively. The small focal spot was used for imaging and large one for radiation treatment. The a-Si detector had a 20 cm × 20 cm detection and 200 µm pixel pitch, and can sustain a frame rate of 25 Hz at full resolution.

### System Calibration

#### CCD *Camera* Calibration

Spatial Distortion: This was examined by imaging a grid board marked with crosslines. Coordinates of the crosses on the image of the grid board were calculated using those at the four corners as references and then compared to their true values.

Uniformity Correction: Ideally, the uniformity correction map can be generated with a large-size integrating sphere that can cover the entire lens with its light output port^61^. Since the integrating sphere was not available in our lab, we developed a special uniformity measurement procedure which requires moving a constant light output across the FOV. The laser at 856 nm was used to provide a constant light output of 1.55 ± 0.05 mW. The cuboid phantom was used to attenuate the laser light and create a light distribution appropriate for intensity measurement. The non-uniformity map was acquired only for one quadrant of the image plane since the non-uniformity pattern should be symmetrical along both horizontal and vertical directions. The phantom and light output were moved together 5 mm stepwise vertically or horizontally until one entire quadrant of the FOV was covered. The peak intensity from each image projection was extracted and plotted in the image coordinates. The light intensity of the remaining pixels within the measured quadrant was interpolated using quadratic curve fitting by fixing the maximal intensity at the image center. A full intensity map was then created by symmetrically mapping this quadrant to the entire FOV. The uniformity correction map was created by normalizing the intensity of each pixel to that of the central pixel.

Image Offset Correction: The dark current and readout noise was measured by averaging 10 images obtained with lens cap blocking the background light. Then the total offset was acquired by averaging another 10 images captured in the dark enclosure without lens cap. The same exposure time 0.02 s as in SPROI/SPROT acquisition was used. The intensity of the background light in the dark enclosure was obtained by subtracting the readout noise and dark current from the total offset.

CCD Reading Linearity: This was checked by acquiring a series of images at different exposure levels at 856 nm. The cylindrical phantom was centered on the beam path to attenuate and diffuse the laser light (0.35 ± 0.05 mW). The mean value of the light intensity within a ROI drawn at the center of the light field was obtained to plot the CCD response curve.

### Light Fluence Calibration

The signal intensity in a CCD image needs be converted to the true light fluence at the object surface for image reconstruction. The lower half of the cylindrical phantom, which was homogeneous, was used to diffuse the laser light and generate a light distribution. The mean light intensity within a 2 × 2 mm^2^ ROI located at the center of the image was measured and averaged over 8 repetitions. Meanwhile, light transmission in the phantom was simulated in NIRFAST with the light source positioned on the surface. The same measurement procedure was repeated where the simulated light fluence was directly obtained from the phantom surface. The ratio of simulated light fluence to measured image intensity was used as a normalization factor to convert CCD signal to light fluence used in NIRFAST. In following experiments, signal intensity was converted to light fluence by multiplication of the normalization factor for all CCD images.

### Imaging in the Phantom

GNR colloid of 0.17 mL (96.3 nM, or 3.57 mg/g) was deposited into the hole of the cylindrical phantom as imaging target. XCT was performed with the 1.0 mm focal spot, 45 kVp, 5.5 mA, and a 0.1 mm thick copper filter. It was reconstructed with 360 x -ray projections acquired one projection per 1° rotation, using the Feldkamp-Davis-Kress (FDK) filtered back projection algorithm^69^. The voxel resolution was 0.13 × 0.13 × 0.13 mm^3^. The XCT was used to create a 3D mesh required for the following SPROT reconstruction, and to confirm the location of GNR within the phantom. For SPROT, optical projections were acquired with the 856 nm laser (14.05 ± 0.05 mW) positioned at 5 different elevations (3 mm apart) and 8 angles (every 45° rotation) per elevation, resulting in a total of 40 images. The images first underwent background and uniformity correction. Then 7 × 7 data points equally spaced were extracted from the central area (22 mm × 18 mm on the imaging plan) of the light distribution in each image. Therefore there were 40 source locations and 49 detector points per source location, resulting in 1960 light-transmission data points. Point intensity was converted to light fluence at phantom surface by multiplication of the normalization factor. Point location was mapped onto surface of phantom XCT, using the geometrical registration method proposed by Yang *et al*.^61^. To reduce time, only the middle part (34 mm length) of the cylinder was used in SPROT reconstruction. A 3D mesh (111280 tetrahedrons, 20108 nodes and 1 mm node-to-node distance) was generated from phantom XCT using a software platform NIRVIEW (Kitware, Inc., Clifton Park, New York, USA). Reconstruction input data include location of detector points, location of light sources, the 3D mesh, which were all presented in the XCT coordinates, as well as surface light fluence. The SPROT reconstruction took 1.3 hours on a computer with a 3.4 GHz CPU and 16 GB RAM.

### Imaging in the Animal

All animal experiments were performed in accordance with the experimental protocol approved by the Institutional Animal Care and Use Committee at the University of Miami. Four 11-week old female nude mice received implantation of 10,000 4T1 cancer cells each into fat pad of the right breast to develop breast carcinoma. Two weeks later, the animals were anesthetized with 20 μl ketamine:xylazine cocktail (100:10 mg/kg body weight) and immobilized on an animal holder for SPROI imaging. Three mice received an intra-tumor injection of 40 μl GNR colloid (46.5 nM, 1.75 mg/g) while one received equal volume of PBS as control. Optical projections of the anterior animal surface were acquired while the 856 nm laser (60.00 ± 0.20 mW) was positioned at the center of mouse lower back. Imaging was performed before and immediately after GNR/PBS administration. All tumors were harvested after imaging and processed for ICP-MS analysis of gold concentration.

The 3D SPROT imaging was performed on an additional mouse bearing breast carcinoma. The XCT imaging parameters were same as the phantom imaging except that the large x-ray focus was used instead. Optical projections of the anterior and posterior animal surface, respectively, were acquired while the 856 nm laser (60.00 ± 0.20 mW) was positioned sequentially at 5 × 5 grid locations (3 mm separation between neighboring locations). Imaging was performed before and immediately after GNR administration. A fluorescence image was also acquired 2 hours after GNR injection to confirm the existence of GNR at the tumor site.

**Figure 8:**
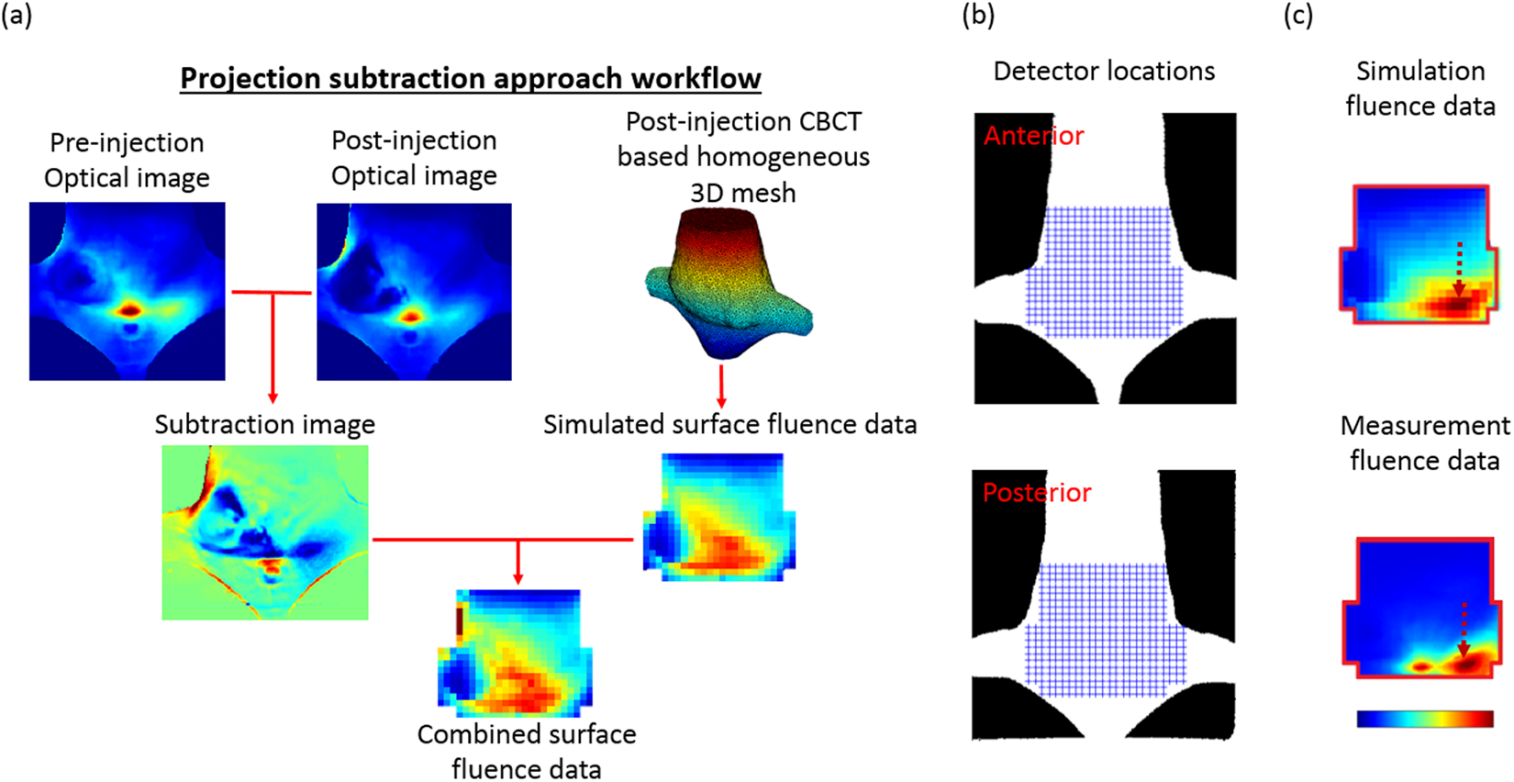
Data process for SPROT reconstruction. (**a**) The post- and pre-injection projection subtraction approach. The calibrated post- and pre-injection optical images were registered and subtracted. This process suppresses the artifact otherwise caused by the high intrinsic absorption from the mouse abdomen. The surface fluence data was simulated based on a homogeneous 3D mesh generated from the XCT, and then added to the subtraction image to form the combined surface fluence data. The combined data was used for SPROT reconstruction. (**b**) The array of detector locations used in SPROT reconstruction. Each cross represents a detector point. (**c**) Comparison of the simulated and measured surface fluence data demonstrates a similar light distribution pattern. The arrows point to the high intensity regions which appeared in the same location. The normalization factor was calculated as the ratio of simulation to measurement data. The redline outline indicates the region of useful detector data.

Workflow of optical data process is shown in Fig. 8(a). Optical images acquired post- and pre- GNR administration were registered and subtracted to suppress the imaging artifacts otherwise caused by the strong light absorption in the bowel. 425 data points (20 rows and 19~24 points per row, 1 mm space, see Fig. 2(b)) were extracted from each subtraction image. Data point was abandoned if its signal intensity was saturated. In total, 50 source locations and 20814 detector points were extracted for reconstruction. A homogeneous 3D mesh was generated based on pre-injection XCT scan and was used to simulate propagated surface fluence data. Simulated surface fluence data was then compared with its correspondent projection image to validate the agreement between the simulation and measurement in light distribution (see Fig. 8(c)). It was also used to calculate the normalization factor. For SPROT reconstruction, a 3D mesh of the mouse lower body (1 mm node-to-node distance, 30956 tetrahedrons and 6008 nodes) was generated based on the post-injection XCT. The light fluence obtained from a simulated forward propagation was added to the normalized subtraction data. The combined fluence data was eventually used for reconstruction.

The tumor was contoured in x-ray and SPROI images both pre- and post-injection. The mean signal intensity within the tumor contour was obtained for statistical analysis. Student’s t-test was used to compare the signal difference between pre- and post- injection images, with the significance determined at p < 0.05.

### ICP-MS analysis

The tumor was rinsed with Milli-Q water (Millipore Corp., Merck KGaA, Darmstadt, Germany, resistivity > 18 MΩ) inside a class-100 Trace Metal Workstation (Microzone Corp., Ottawa, Ontario, Canada). The samples were weighed onto 30 mL fluorinated ethylene propylene (FEP) vials that were pre-cleaned in 3 mol/L HNO_3_ and boiling Aqua Regia (HCl:HNO_3_ at 3:1). The acid solutions were distilled once from ACS grade HCl and HNO_3_ in DST-1000 sub-boiling stills (Savillex Corp., Eden Prairie, MN, USA). Over the course of three weeks, the samples range between 0.2~1.6 g were dissolved in approximately 20 g of Aqua Regia. The vials were heated on a hotplate at 180 °C for 5 days followed by 30 minutes of vortex-assisted ultrasonication and agitation at 6000 RPM and the cycle was repeated 3 more times. The solutions containing the fully dissolved tissues were diluted gravimetrically by adding Milli-Q water to a total 50 g. Approximately 0.05 g of this solution was further diluted by a factor of 20 and then analyzed on a Neptune Plus Multi-Collector Inductively Coupled Plasma Mass Spectrometer (MC-ICP-MS)^70^. The concentration of gold in tissue was determined by standard-sample bracketing technique against a certified multi-element standard solution of 10 µg mL^−1^ of Au, Ir, Pd, Pt, Re, Rh, Ru and Te. The sample and the standard solutions were introduced to the mass spectrometer in 0.45 mol L^−1^ HCL/HNO_3_ using a self-aspirating nebulizer (nominal flow of 400 µL min^−1^).

Data acquisition involved three blocks of three cycles with 4.194 s integration time on the plateau of the ^197^Au ion beam as measured in the center faraday detector. Sample uptake time was 70 s. Baseline calibration was performed prior to each data acquisition. The sample was measured three times and the concentration of Au in the running solution was quantified relative to the certified standard solution according to the following relationship:

1 $${C}_{A}={C}_{S}\times \frac{{I}_{A}}{{I}_{S}}$$

where C_A_ and C_S_ represent the concentrations of Au, and I_A_ and I_S_ represent the intensities of the Au ion beam, in the sample and standard solutions, respectively^70^. The measurement produced a signal intensity of 0.35 V in a solution of 200 ng g^−1^ of Au.

### SPROT Reconstruction

SPROT reconstruction was performed in NIRFAST with customized optimization algorithm. Based on finite element method (FEM), NIRFAST solves the inverse problem by solving the Tikhonov minimization^67,71–73^

2 $${\rm{\Omega }}=\mathop{\min }\limits_{\mu }\{\sum _{i=1}^{NM}{({{\rm{\Phi }}}_{i}^{M}-{{\rm{\Phi }}}_{i}^{C})}^{2}+\lambda \sum _{j=1}^{NN}{({{\rm{\mu }}}_{j}-{{\rm{\mu }}}_{0})}^{2}\}$$

iteratively with

3 $${({J}^{T}J+2\lambda I)}^{-1}{J}^{T}\delta {\rm{\Phi }}=\delta \mu $$

when the Jacobian matrix is overdetermined, or with

4 $${J}^{T}{(J{J}^{T}+2\lambda I)}^{-1}\delta {\rm{\Phi }}=\delta \mu $$

when the Jacobian matrix is underdetermined. *NM* is the number of detector measurements, *NN* is the number of unknown parameters of optical properties, ${{\rm{\Phi }}}_{i}^{M}$ is the detector measurements, ${{\rm{\Phi }}}_{i}^{C}$ is the detector data calculated from the forward model, μ_0_ is the initial guessing of the unknown parameters, *λ* is the Tikhonov regularization parameter, *J* is the Jacobian matrix, *I* is the identity matrix, *δ*Φ is the data-model misfit $({{\rm{\Phi }}}_{i}^{M}-{{\rm{\Phi }}}_{i}^{C})$ and *δμ* is the iterative optical property update.

The initial values of u_a_ and u^’^_s_ were set to 0.01 mm^−1^ and 1.00 mm^−1^, representing the optical properties of adipose tissue in the abdomen. After each iteration, nodes with absorption coefficients ≥0.1 mm^−1^ (~10 times of soft tissue u_a_) were recognized as nodes containing GNR, and the remaining nodes were recognized as background with zero GNR concentration. For the gold-containing nodes, the u_a_ were optimized and u^’^_s_ updated iteratively following a known relationship between u_a_ and u^’^_s_ for the specific GNR used (calculated absorption to scattering ratio of about 50). For the background nodes, their u^’^_s_ were reset to the initial values. Jacobian normalization was implemented by dividing each column of the Jacobian matrix with this column’s Euclidean norm

5 $${f}_{j}=\sqrt{\sum _{i=1}^{NM}{{J}_{i,j}}^{2}}$$

at the beginning of each iteration. Here *i* and *j* are the row and column number of the Jacobian matrix, respectively. This procedure was to normalize the sensitivity of nodes and result in a smoother distribution of reconstructed optical properties.

The datasets generated during and/or analyzed during the current study are available from the corresponding author on reasonable request.
